# EHealth Acceptance and New Media Preferences for Therapy Assistance Among Breast Cancer Patients

**DOI:** 10.2196/cancer.5711

**Published:** 2016-09-14

**Authors:** Caroline Drewes, Thomas Kirkovits, Daniel Schiltz, Timo Schinkoethe, Renate Haidinger, Ursula Goldmann-Posch, Nadia Harbeck, Rachel Wuerstlein

**Affiliations:** ^1^ Breast Center, University Hospital Munich Department of Gynecology and Obstetrics, CCC of LMU Munich Germany; ^2^ Brustkrebs Deutschland e.V. Munich, Ulm Germany; ^3^ Mamazone e.V. Munich, Augsburg Germany

**Keywords:** eHealth, mHealth, breast cancer, adherence, compliance, new media, therapy improvement

## Abstract

**Background:**

Electronic health (eHealth) and mobile communication-based health care (mHealth) applications have been increasingly utilized in medicine over the last decade, and have facilitated improved adherence to therapy regimens in patients with chronic conditions. Due to the long duration of breast cancer therapy, and the long course of disease in metastatic breast cancer, a need for more intensified physician-patient communication has emerged. Various support mechanisms, including new media such as mHealth and eHealth, have been proposed for this purpose.

**Objective:**

The aim of this study was to analyze the correlation between sociodemographic factors, as well as health status of breast cancer patients, and their current utilization of new media, or their willingness to use Internet and mobile phone apps for improvement of therapy management.

**Methods:**

The survey for this study was conducted anonymously during the 2012 Mamazone Projekt Diplompatient meeting (Augsburg, Germany), which hosted approximately 375 participants per day. A total of 168 questionnaires were completed. The questionnaire aimed to assess sociodemographic status, disease patterns, and current use of new media (ie, Internet, mobile phone, and mobile phone apps) in breast cancer patients. Habits and frequency of use for these new technologies, as well as patients’ affinity towards eHealth and mHealth tools for therapy management improvement, were investigated.

**Results:**

Almost all participants used the Internet (95.8%, 161/168), with 91.5% (151/165) also utilizing this technology for health-related issues. Approximately 23% (38/168) of respondents owned a mobile phone. When asked about their preferences for therapy assistance, 67.3% (113/168) of respondents were interested in assistance via the Internet, 25.0% (42/168) via mobile phone, and 73.2% (123/168) via call center. Patients diagnosed with breast cancer <5 years before the survey were significantly more interested in a call center than patients diagnosed >5 years before survey participation.

**Conclusions:**

The vast majority of breast cancer patients accept the Internet for therapy assistance, which indicates that eHealth is a promising medium to improve patient-physician communication. Such technologies may improve individual disease management and ultimately lead to an enhanced adherence to therapy regimens.

## Introduction

Breast cancer is the most common cancer among women, with a worldwide incidence of 1.4 million in 2008 [1]. In Germany, the mean age of breast cancer patients is 65 years, but 25% of newly diagnosed women in 2008 were younger than 55 years of age [2]. Due to early diagnoses and therapy advances such as adjuvant endocrine therapy, breast cancer survival rates have increased [3]. Patients with potentially curable breast cancer may be viewed as having a chronic disease, due to adverse treatment effects, other co-morbidities, and the burden of having a life-threatening disease that might recur [4]. Endocrine agents are useful breast cancer treatments, but therapy duration is crucial for optimal treatment benefit [5]. A study by Hadji et al revealed that breast cancer patients with a poor compliance to drug regimens are at high risk for early treatment discontinuation [6]. Thus, despite improved survival rates, breast cancer patients are still at risk for cancer recurrence [7], partly due to improper therapy usage. The long duration of adjuvant therapies may lead to diminished patient adherence (the World Health Organization Adherence Meeting defined *adherence* as the extent to which the patient follows the prescribed instructions [8]) and thus poor health outcomes. Increased adherence may lead to better health outcomes and decreased health care costs [8].

New technologies offer promising strategies to reduce treatment nonadherence. The Internet has become increasingly important and relevant for health-related purposes. In 2013, the Federal Statistical Office estimated that 79% of the population had Internet access in Germany, and Internet use had increased from 65% in 2006 to 79% in 2013 [9]. Another survey stated that almost two thirds of German Internet users search for health information online [10]. Kummervold et al reported that the sources that patients use for health information have changed (with a transition to the Internet), and consequently there was a decrease in patients contacting health professionals [11]. Rozenblum states that the Internet has become a powerful tool for communication and involvement of patients in their own health care [12].

Fogel and colleagues demonstrated a supportive benefit among Internet users with breast cancer [13], leading to more individual responsibility. Patients feeling insecure or overwhelmed during their face-to-face visits can better concentrate, and feel more motivated to ask questions or retrieve information, when communicating by email [14]. Breast cancer diagnoses often cause differing levels of depression, anxiety, and distress (even years after the initial diagnosis), necessitating further patient support [15]. In addition, by providing clear and accurate informative websites, breast cancer patients feel more prepared for their diagnoses [16]. Ybarra et al described the influence of health-related websites as an important influence on patient behavior, leading to less anxiety and increased self-efficacy [17].

Another effect that can be facilitated by new media is *self-management*. Self-management of patients leads to a more actively involved patient population and positively affects chronic disease management [18,19]. However, the prospective Patients' Anastrozole Compliance to Therapy study demonstrated that the mere provision of educational material (without an interactive component) did not significantly improve compliance with aromatase inhibitor therapy in postmenopausal women with early, hormone receptor-positive, breast cancer [20]. Nevertheless, patients who truly understand their therapy concept may better adopt their care plans, and thus have enhanced adherence, if a more interactive approach is taken. The Internet already plays an established role in such interventions by conveying personalized messages [21]. Furthermore, mobile communication-based health care (mHealth) represents a highly developed tool, and another way to meet the challenges encountered in medicine. In Germany, 44.0 million people owned a mobile phone in 2015 [22], and according to an American survey, 25% of all mobile phone owners already use health care apps [23]. In order to monitor adherence, Morak et al proposed to record the intake of prescribed medication via mobile phone apps [24]. In a review, Fiordelli et al stated that the number of articles discussing mHealth has substantially increased over the last 5 years, and that the main focus of mHealth research is chronic conditions [25].

New technologies may provide an opportunity to improve physician-patient communication and secure better data exchanges. Furthermore, patient education and self-management may be achieved using electronic health (eHealth) and mHealth, eventually leading to better clinical outcomes. While many studies have already analyzed nonadherence in chronic diseases, few have focused on factors influencing breast cancer patients’ affinity towards modern technologies. This study aims to identify breast cancer patients’ sociodemographic and health factors influencing their affinity towards new media (ie, Internet, mobile phone apps, call centers), and their willingness to use such technologies for health-related problems.

## Methods

### Questionnaire

A German-language questionnaire was developed with the support of Mamazone and Brustkrebs Deutschland, two large German breast cancer advocacy groups. The questionnaire consisted of 33 items. To prevent any selection bias, the questionnaire was designed as a paper-based handout. This study was approved by the local ethics committee of the Ludwig Maximilian University. The questionnaire contained four parts: closed questions with different choices (parts 1, 2, and 4) and questions with multiple possible answers (part 3).

Part 1 included six sociodemographic items regarding age, sex, residential area (by postal code) and population, number of people per household, education, and employment. Education was divided into either junior high school (9 or 11 years of school attendance) or senior high school (13 years of school attendance). Patients with a university or doctoral degree were added to the group of patients who had a senior high school degree, since a senior high school diploma is a prerequisite for university studies or a doctoral degree.

Part 2 focused on the patient’s health condition by examining the following parameters: Eastern Cooperative Oncology Group (ECOG) score, diagnosis of breast cancer, time since initial diagnosis, metastatic status, therapy, diagnosis of other cancer, and menopausal status. ECOG was used to measure patients' current and subjective well-being and disease-related impairment of their daily life. The measure ranges from 0 to 5 (0=no restrictions from disease, 1=restriction in physically strenuous activity, 2=capable of self-care but unable to carry out work activities, 3=capable of only limited self-care, 4=incapable of self-care, and 5=deceased) [26].

Part 3 assessed the frequency of technology usage, including mobile phones, computers, the Internet, and apps via questions such as, “ *Which types of electronic equipment do you possess: telephone, computer?* ” Mobile phone and Internet habits were also examined.

Part 4 measured patients' interest in future interactions with new media. This section assessed patients’ interest in purchasing a mobile phone for health support in general, the acceptance of therapy assistance via the Internet and/or mobile phone, the approval of the Internet and/or mobile phone for side effect documentation, and the acceptance of call centers for support (call centers that contact patients to ask for their well-being vs call centers that automatically transfer information to the physician). Answers for this part were rated from 1 to 5 (1=the highest acceptance, 2=high acceptance, 3=a neutral position, 4=low approval, and 5=no acceptance).

### Participants

The survey was conducted during the 2012 Mamazone Projekt Diplompatientin meeting in Augsburg, Germany [27]. This meeting involved advanced training for breast cancer patients (and physicians) and took place over 4 days, hosting an average of 375 daily participants. The paper-based questionnaire was handed out on one day with 393 attendants (one questionnaire each), and was completed by the respondents during the meeting. Participation was voluntary and anonymous. There was no prior selection concerning sex, age, or ethnic groups.

### Statistical Analyses

Descriptive statistics (ie, frequency, mean, and median) were used to characterize user patterns. In order to better understand patient preferences towards new media, a univariate analysis was used to explore demographic factors associated with certain response types. Odds ratios were used to compare the strength of the correlation between acceptance of new media usage and potential predictors. With the help of logistic regression, the odds ratios between groups were calculated, with a 95% CI. A *P* value of <.05 indicated statistical significance. We analyzed the data using IBM SPSS version 22 for statistical calculations.

## Results

The questionnaire was completed by 168 of 393 participants at the Mamazone Projekt Diplompatientin meeting (return rate of 42.7%). Some questions remained unanswered on otherwise completed questionnaires (Table 1).

### Part 1: Sociodemographic Facts

The majority of the participants were female (98.2%, 164/167) with a median age of 56.0 years (range 28-76 years). A small proportion of participants (3.9%, 6/154) were younger than 40 years, 12.3% (19/154) were between 40-50 years, 49.3% (76/154) were between 50-60 years, 26.0% (40/154) were between 60-70 years, and 8.4% (13/154) were older than 70 years. Approximately 26.7% (43/161) of patients lived in a household with at least three people, 44.7% (72/161) lived just with their partners, and 28.6% (46/161) lived alone. A senior high school degree was accomplished by 66.5% (111/167) of participants, and 33.5% (56/167) had graduated from junior high school. Approximately one third (32.9%, 53/161) were pensioners, 41.6% (67/161) were employed, 11.8% (19/161) were self-employed, and 1.2% (2/161) were unemployed (Table 1).

### Part 2: Patients’ Health

Most participants (97.0%, 163/168) identified themselves as breast cancer patients. Approximately half of all patients had suffered from breast cancer for more than five years (46.0%, 75/163), while 15.3% (25/163) were confronted with first diagnosis within the previous year. Furthermore, 25.6% (43/161) already had metastatic disease and 74.8% (119/159) were postmenopausal. More than two thirds of respondents had intravenous chemotherapy (72.6%, 122/168) and 9.5% (16/168) had undergone oral chemotherapy. Most participants (75.0%, 126/168) had undergone anti-hormone therapy and 22.0% (37/168) underwent antibody therapy. Almost all patients (97.6%, 164/168) had undergone an operation and 81.0% (136/168) had received radiation therapy. ECOG 0 and 1 were the most common answers when patients were asked about their physical status.

### Part 3: Use of Media

Most participants (95.8%, 161/168) used the Internet. Table 2 outlines the reasons why, and how often, respondents searched the Internet. Multiple answers were allowed. The majority of participants used the Internet daily (61.3%, 103/168) or at least once a week (26.8%, 45/168), with 4.2% (7/168) rarely or never going online. The majority of Internet users (88.1%, 148/168) indicated that the purpose of their use was for reading or sending emails, and 53.0% (89/168) confirmed the use of online encyclopedias. In terms of web-based communication, 26.8% (45/168) of the participants expressed their affinity towards social networks and 16.1% (27/168) of the participants expressed their opinions online. Similarly, 92.1% (139/151) of patients with health-related Internet use specified their search interests. The performed tasks included: seeking general information about breast cancer (92.7%, 140/151), searching for information about physicians/hospitals (66.9%, 101/151), contacting physicians (12.6%, 19/151) or pharmacists (0.7%, 1/151), exchanging information with other patients (34.4%, 52/151), and searching for therapies (64.2%, 97/151) or scientific information (68.2%, 103/151). Approximately 9.8% (15/153) of the participants indicated mobile phone usage for health-related issues.

**Table 1:** Patient characteristics.

		%	N (amount/total)
**Total**		168
**Gender**			
	Female	98.2	164/167
	Male	1.8	3/167
**Age in years, mean/median (range)**		54.6/56.0 (28-76)
**Education**			
	Junior high school and below	33.5	56/167
	Senior high school and above	66.5	111/167
**Residents in the local community**			
	<1000	7.9	13/165
	1000-9999	19.4	32/165
	10,000-49,999	29.7	49/165
	50,000-99,999	5.5	9/165
	>100,000	37.6	62/165
**Employment**			
	Unemployed	1.2	2/161
	Official	12.4	20/161
	Employed	41.6	67/161
	Self-Employed	11.8	19/161
	Pensioner	32.9	53/161
**Number of people per household**			
	1	28.6	46/161
	2	44.7	72/161
	3	13.7	22/161
	>4	13.0	21/161
**Diagnosed with breast cancer**		97.0	163/168
**Time since onset**			
	Last month	0.6	1/163
	Last year	14.7	24/163
	1-5 years ago	38.7	63/163
	>5 years ago	46.0	75/163
**Metastatic disease**		25.6	43/161
**Therapies**			
	Operation	97.6	164/168
	Chemotherapy - intravenous	72.6	122/168
	Chemotherapy - oral	9.5	16/168
	Anti-hormonal therapy	75.0	126/168
	Antibody therapy	22.0	37/168
	Radiation	81.0	136/168
	Other therapy	17.9	30/168
**Menopausal status**		74.8	119/159
**Other cancer**		7.3	12/165
**ECOG**			
	0	71.2	116/163
	1	25.2	41/163
	2	3.7	6/163

**Table 2:** Internet usage by patients with breast cancer.

		%	N (amount/total)
**Internet use in general**		95.8	161/168
**Frequency of Internet use**			
	Daily	61.3	103/168
	>1/week	26.8	45/168
	>1/month	3.6	6/168
	Rarely/never	4.2	7/168
**Types of Internet use**			
	Email	88.1	148/168
	Social networks	26.8	45/168
	Reading online news, articles	48.2	81/168
	Usage of Wikis/online encyclopedia	53.0	89/168
	Search for information about products/services	54.2	91/168
	Read/express opinions on the web	16.1	27/168
	Participation in counseling/vote (ie, city planning)	8.9	15/168
	Participation in online courses for private education/qualification	5.4	9/168
**Internet use for health-related issues**			
	General information about my disease	92.7	140/151
	Search for information about physicians/hospitals	66.9	101/151
	Contact my physician	12.6	19/151
	Contact my pharmacist	0.7	1/151
	Exchanging information with other patients	34.4	52/151
	Search for therapies	64.2	97/151
	Scientific information	68.2	103/151
**Mobile phone use for health-related purposes**		9.8	15/153

Internet and mobile phone usage was examined, as detailed in Table 3. All participants (100.0%, 25/25) up to the age of 50 used the Internet on a regular basis, and 81.8% (9/11) of the participants older than 70 years used the Internet for health-related issues. All participants (100.0%, 21/21) living in a household with more than 4 people, and 89.1% (41/46) of the participants who lived alone, were Internet users. Moreover, 97.3% (108/111) of the participants with a senior high school degree used the Internet and 93.6% (102/109) used it for health-related purposes. Most participants with a junior high school degree used the Internet (92.9%, 52/56) and many used it for health-related purposes (87.3%, 48/55). There was no apparent correlation between place of residence or employment and the use of new media. When examining medical patient characteristics, 96.0% (24/25) of the patients diagnosed with breast cancer within the last year had already searched the Internet for health-related issues, along with 77.3% (58/75) of those diagnosed >5 years prior. Approximately 95.1% (39/41) of patients with metastatic breast cancer and 91.1% (102/112) of patients with a postmenopausal status searched the Internet for health inquiries. There was no correlation between therapies and Internet use.

**Table 3:** Comparison of Internet and mobile phone usage by patients with breast cancer.

Characteristics		Internet use in general % (n)	Internet use for health-related issues % (n)	Mobile phone owner % (n)
**Total**		95.8 (161/168)	91.5 (151/165)	22.6 (38/168)
**Age (years)**				
	<39	100.0 (6/6)	100.0 (6/6)	50.0 (3/6)
	40-49	100.0 (19/19)	100.0 (29/29)	31.0 (9/29)
	50-59	98.7 (75/76)	90.8 (69/76)	19.7 (15/76)
	60-69	97.5 (39/40)	89.7 (35/39)	20.0(8/40)
	>70	61.5 (8/13)	81.8 (9/11)	15.4 (2/13)
**People in household**				
	1	89.1 (41/46)	88.4 (38/43)	13.0 (6/46)
	2	97.2 (70/72)	91.7 (66/72)	26.4 (19/72)
	3	100.0 (22/22)	100.0 (22/22)	36.4 (8/22)
	>4	100.0 (21/21)	95.2 (20/21)	19.0 (4/21)
**Education**				
	Junior high school and below	92.9 (52/56)	87.3 (48/55)	17.9 (10/56)
	Senior high school and above	97.3 (108/111)	93.6 (102/109)	25.2 (28/111)
**Employment**				
	Unemployed	100.0 (2/2)	100.0 (2/2)	0.0 (0/2)
	Official	95 (19/20)	94.7 (18/19)	20.0 (4/20)
	Employed	97.0 (65/67)	91.0 (61/67)	17.9 (12/67)
	Self-employed	100.0 (19/19)	100.0 (19/19)	36.8 (7/19)
	Pensioner	92.5 (49/53)	88.2 (45/51)	20.8 (11/53)
**Residents in the local community**				
	<1000	100.0 (13/13)	92.3 (12/13)	23.1 (3/13)
	1000-9999	96.9 (31/32)	96.9 (31/32)	34.4 (11/32)
	10,000-49,999	98.0 (48/49)	91.7 (44/48)	22.4 (11/49)
	50,000-99,999	88.9 (8/9)	100.0 (9/9)	33.3 (3/9)
	>100,000	93.6 (58/62)	86.7 (52/60)	14.5 (9/62)
**Diagnosed breast cancer**		92.3 (144/156)	85.9 (140/163)	22.1 (36/163)
**Time since onset**				
	Last month	100.0 (1/1)	100.0 (1/1)	0.0 (0/1)
	Last year	91.7 (22/24)	95.8 (23/24)	20.8 (5/24)
	1-5 years ago	95.0 (57/60)	92.1 (58/63)	22.2 (14/63)
	>5 years ago	90.1 (64/71)	77.3 (58/75)	22.7 (17/75)
**Metastatic disease**		95.1 (39/41)	90.7 (39/43)	27.9 (12/43)
**Therapies**				
	Operation	92.4 (145/157)	86.0 (141/164)	22.0 (36/164)
	Chemotherapy - intravenous	93.2 (110/118)	87.7 (107/122)	26.2 (32/122)
	Chemotherapy - oral	93.8 (15/16)	87.5 (14/16)	31.3 (5/16)
	Anti-hormonal therapy	90.9 (110/121)	83.3 (105/126)	21.4 (27/126)
	Antibody therapy	94.4 (34/36)	97.3 (36/37)	24.3 (9/37)
	Radiation	93.0 (120/129)	85.3 (116/136)	21.3 (29/136)
	Other therapy	93.1 (27/29)	86.7 (26/30)	26.7 (8/30)
**Menopausal status**		91.1 (102/112)	83.2 (99/119)	17.6 (21/119)
**Other cancer**		81.8 (9/11)	75.0 (9/12)	16.7 (2/12)

Almost one fourth of participants (22.6%, 38/168) owned a mobile phone, and younger participants were more likely to use this technology. Furthermore, 17.9% (10/56) of participants with a junior high school degree owned a mobile phone, along with 25.2% (28/111) of the participants with a senior high school degree. The highest ratio of mobile phone ownership was observed in self-employed patients (36.8%, 7/19), followed by pensioners (20.8%, 11/53) and public servants (20.0%, 4/20). Patients’ health status did not correlate with mobile phone ownership.

### Part 4: Patients’ Future Interests

**Figure 1:**
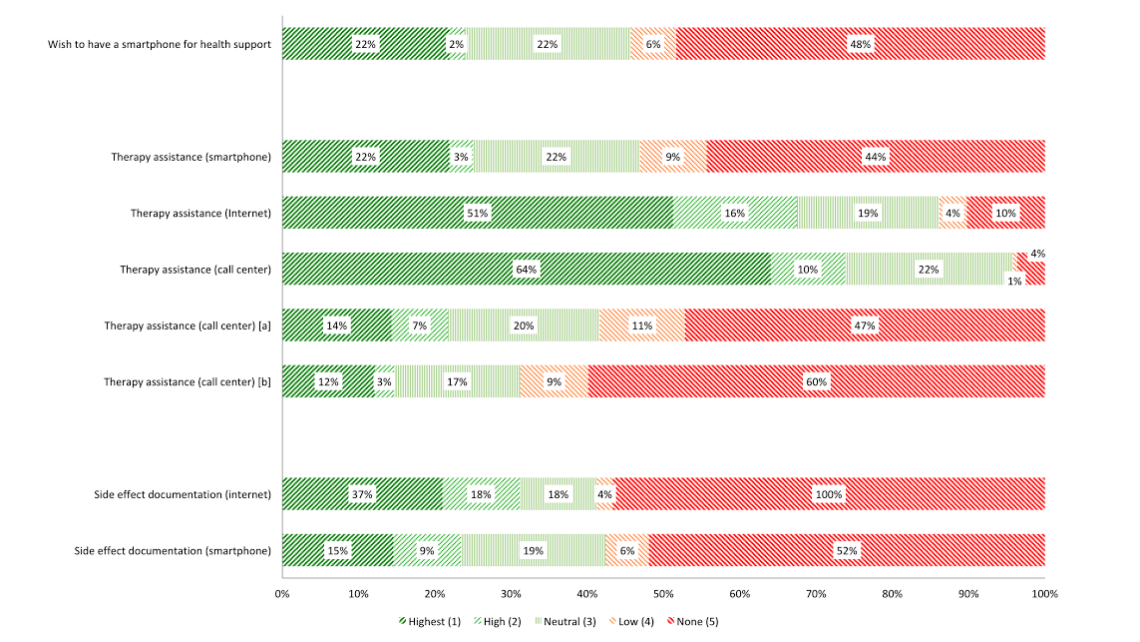
Breast cancer patients’ interests in further interaction with new media. a) those who call and receive information about your condition; b) those who pass forward information to your physician.

Figure 1 shows respondents’ acceptance towards various types of communication. The affinity towards each technology is represented by different colors (green=high acceptance; light green, straight lines= a neutral position; red= negative views or disapproval). A call center that a patient can contact for therapy support was acceptable to 73.2% (123/168) of participants, followed by the Internet (67.3%, 113/168), and mobile phones (25.0%, 42/168). Consequently, mobile phones were rejected by 52.9% (89/168) of participants, the Internet by 13.7% (23/168), and the call center by 4.2% (7/168). Approximately one forth of respondents indicated a more neutral position towards each of the three categories. Call centers were approved if they actively called the patients and asked about the patients’ condition (22.0%, 37/168), or if the center passed on information to the patients’ physician (14.9%, 25/168). Furthermore, 54.8% (92/168) of the participants would agree to document their side effects via the Internet, and 23.2% (39/168) would do so via mobile phones.

### Potential Predictors for New Media Use

Odds ratios were used to demonstrate the impact of different factors on the participants’ wishes regarding new media for therapy improvement (Table 4). The median age of patients was 56 years. The group of patients was divided into age ranges in order to analyze the impact of age on the use of each medium (>56 years vs <56 years). A highly significant difference was observed when comparing the acceptance of mobile phones for therapy assistance (*P*<.001) and side effect documentation (*P*=.002) between younger versus older patients. Moreover, therapy assistance via call center (*P*=.001), via the Internet (*P*=.036), and acceptance of side effect documentation with the help of the Internet (*P*=.024), were significantly more likely to be endorsed by younger participants.

**Table 4:** Correlations of patients' characteristics and their desire for new media usage for therapy assistance.

Characteristics		Odds ratios (CI) with *P* values			
		Younger versus older	Multiple versus one-person household	Senior high school versus junior high school graduation	Time since onset <5years versus >5years
**Acceptance of new media**					
	Having a mobile phone	1.79 (0.86-3.74) *P*=.084	2.46 (0.95-6.37) *P*=.042	1.57 (0.71-3.55) *P*=.176	1.97 (0.48-2.06) *P*=.567
	Wish to obtain a mobile phone for health support	2.38 (1.06-5.33) *P*=.027	1.65 (0.68-4.02) *P*=.185	1.08 (0.48-2.43) *P*=.515	1.40 (0.63-3.09) *P*=.263
**For therapy assistance**					
	Via mobile phone	4.15 (1.83-9.43) *P*<.001	2.68 (1.05-6.82) *P*=.026	0.96 (0.44-2.08) *P*=.532	1.26 (0.59-2.69) *P*=.342
	Via Internet	2.64 (1.01-6.91) *P*=.036	2.78 (1.04-7.47) *P*=.039	1.17 (0.46-3.02) *P*=.458	2.50 (0.98-6.38) *P*=.042
	Via call center	3.59 (1.66-7.77) *P*=.001	0.79 (0.35-1.81) *P*=.369	1.09 (0.51-2.34) *P*=.489	3.50 (1.62-7.55) *P*=.001
**For side effect documentation**					
	Via Internet	2.24 (1.08-4.72) *P*=.024	1.68 (0.82-4.22) *P*=.103	1.02 (0.47-2.18) *P*=.557	1.85 (0.89-3.84) *P*=.07
	Via mobile phone	3.45 (1.52-7.83) *P*=.002	1.91 (0.77-4.71) *P*=.114	1.05 (0.47-2.34) *P*=.536	0.97 (0.45-2.08) *P*=.54

Patients with a multiple-person household were significantly more often in possession of a mobile phone (*P*=.042), and more likely to accept therapy assistance via mobile phone (*P*=.026) or via the Internet (*P*=.039) compared to patients living alone. No trend was observed when comparing the acceptance of therapy assistance via call center (*P*=.369), or side effect documentation via the Internet (*P*=.103) or mobile phone (*P*=.114), when comparing household sizes.

Participants with a senior high school degree (compared to those with a junior high school degree) showed no significant difference when asked about their acceptance of new media for therapy assistance (mobile phone *P*=.532; Internet *P*=.458; call center *P*=.489). Moreover, participants with a senior high school degree were not significantly more often in possession of a mobile phone (*P*=.176), and do not accept mobile phones (*P*=.557) or the Internet (*P*=.536) more frequently for side effect documentation, than those with junior high school degrees.

When correlating the time since cancer onset with the desire for therapy assistance via new media, patients diagnosed <5 years prior were significantly more interested in therapy assistance via call center (*P*=.001) and the Internet (*P*=.042) than those diagnosed earlier. No significance was observed between the time since disease diagnosis and the acceptance of side effect documentation via the Internet (*P*=.07) or mobile phones (*P*=.54).

## Discussion

The intention of our study was to examine the current use of computers, the Internet, and mobile phones among breast cancer patients, as well as their acceptance towards telecommunication with health care providers. Patients who took part in the survey were very well informed (so-called *diploma* patients); this population was chosen intentionally, as eHealth is a relatively modern issue. The study questionnaire was handed out at a meeting with a full schedule of lectures, so a relatively low response rate was expected. Of the 168 participants, five stated that they did not have breast cancer, which may be due to mistakes, or because family or friends of patients completed the questionnaire.

We compared different sociodemographic and health care factors to technology usage. Future therapy-assistance interventions via new media could be focused according to patient characteristics. Although surveys have already investigated patient characteristics that may affect Internet affinity, this study entailed the first survey focusing specifically on breast cancer patients. It is unclear how many breast cancer patients show Internet affinity, or what their preferences towards new electronic devices actually are.

Our results demonstrate that 86.3% (145/168) of breast cancer patients have at least a neutral opinion towards using the Internet for therapy assistance, while 67.3% (113/168) highly approve it. Moreover, 54.8% (92/168) of patients were willing to document side effects via the Internet. Decisive factors influencing patients’ willingness to use new communication technologies include age, number of people per household, and time since breast cancer diagnosis. Education is not a significant predictor for technology acceptance with regard to therapy improvement.

Considering the outcomes of other studies, it is already an established fact that participants using the Internet in general (and for health-related issues in particular) are mostly young people [11,28]. Recently, Internet acceptance has reached an older age group, meaning that patients older than 60 years in our study are already more familiar with the Internet (88.7%, 47/53) than reported by other studies, in which only 41% of people older than 65 years used the Internet [29]. The patients in the current study possessed a particularly high level of interest in their health (*diploma patients*), while the Federal Office for Statistics reports that the general German population consists of healthy and nonhealthy inhabitants. However, comparing peoples’ health-related Internet queries, it appears that 76% of women older than 65 years in the statistics of the Federal Office [29], and 89.7% (35/39) of the women between 60-69 years in our survey, search the Internet for health information. This trend indicates that the Internet is more appealing as a source of health-related information in older populations than general use of the Internet.

Regarding the acceptance of therapy assistance, younger patients were significantly more interested in new forms of communication and documentation of distressful effects via the Internet or mobile phones. Nevertheless, the results of our study demonstrate that participants of all ages have already searched the Internet for therapies and scientific information, along with information about physicians and/or hospitals. This finding indicates that using the Internet for health-related searches is already widespread, and appears to be feasible for most patients. Furthermore, the survey reveals a lower acceptance of communication via mobile phone features (eg, apps), and a significant and substantial difference between older and younger patients’ consent towards therapy assistance via mobile phones. This result is not surprising, considering that in our survey, younger people were more often in possession of a mobile phone than the older patient population. Mobile phones are a medium with a quickly growing number of customers; in December, 2010, 14 million Germans were in possession of a mobile phone, and this number increased to 21 million in December, 2011, and 31 million in December, 2012 [22]. Demographic changes may have already spread further, as the questionnaire was completed in 2012 and Internet connectivity and functionality (and prices of mobile devices) are currently more feasible for consumers.

In summary, age appeared to be a significant factor for determining interest in therapy assistance via new media, but this trend may soon change, as Internet access and computer literacy are increasing in society. This trend agrees with Kummervold et al, who noted that the rate of Internet use for health-related issues in Germany increased from 24% in 2002 to 57% in 2007 [30]. Along with the demographic change, we assume that consumers will adapt to new media and thus use mobile phones more often for health concerns.

The second factor influencing patients’ affinity towards new media was a multiple-person household. Patients living with a partner or with their children might have assistance with such technologies, and appeared to be more familiar with the Internet. In contrast, a call center was similarly accepted by all patients, regardless of how many people they lived with. This medium does not involve computer knowledge.

Comparing the affinity of breast cancer patients to new media and their time since diagnosis, it can be perceived that the longer patients have been living with the diagnosis, the less interested they were in searching the Internet for health-related issues. This effect may be due to the fact that patients living with the diagnosis for several years have already exploited numerous resources (ie, the Internet or support groups). Thus, these patients have already assembled more information than patients who were recently diagnosed with cancer. Moreover, patients diagnosed >5 years prior to answering the questionnaire might be older, and less interested in modern technologies.

### Conclusion

The Internet, as a rapidly growing medium, was used by almost all participants who completed the questionnaire. Not surprisingly, when asked about their willingness to use new technologies for therapy improvement, the Internet was accepted by the majority of patients. However, not all users were interested in using Internet-based applications for therapy improvement. Among those already using the Internet, only two thirds were willing to use it for therapy improvements. The remaining respondents might be unwilling to use the Internet for such purposes due to unknown implementation of the potential applications. The questionnaire did not specify how new media can be used for health care concerns. To overcome this issue, it is necessary to tailor the implementation of eHealth to patients’ individual needs. One approach to address this issue would be to explore the feasibility of new media with patients and physicians in a future survey. It will be important to focus further research on the technical availability and educational content of patient-specific applications. We determined that with the help of new technologies, self-efficacy (and thereby adherence to therapy regimens) may be improved. The results of the survey confirmed the potential of new media (ie, Internet portals or mobile phone apps) to provide continuous patient-physician communication.

## Abbreviations

ECOG: Eastern Cooperative Oncology Group
eHealth: electronic health
mHealth: mobile communication-based health care
